# Cerebrospinal Fluid Biomarkers of Alzheimer's Disease Show Different but Partially Overlapping Profile Compared to Vascular Dementia

**DOI:** 10.3389/fnagi.2017.00289

**Published:** 2017-09-12

**Authors:** Franc Llorens, Matthias Schmitz, Tobias Knipper, Christian Schmidt, Peter Lange, Andre Fischer, Peter Hermann, Inga Zerr

**Affiliations:** ^1^Department of Neurology, Universitätsmedizin Göttingen Göttingen, Germany; ^2^Center for Networked Biomedical Research on Neurodegenerative Diseases Barcelona, Spain; ^3^German Center for Neurodegenerative Diseases–DZNE Site Göttingen Bonn, Germany

**Keywords:** Alzheimer's disease, amyloid beta, biomarkers, cerebrospinal fluid, neurodegeneration, tau, vascular dementia, vascular encephalopathy

## Abstract

Vascular factors increase the risks of developing Alzheimer's disease (AD) and they contribute to AD pathology. Since amyloid beta (Aβ) deposits can be observed in both diseases, there is an overlap which impedes a clear discrimination and difficult clinical diagnosis. In the present study, we compared cerebrospinal fluid (CSF) profiles of neurodegenerative and inflammatory biomarkers in a patient cohort of controls (*n* = 50), AD (*n* = 65) and vascular dementia (VaD) (*n* = 31) cases. Main results were validated in a second cohort composed of AD (*n* = 26), rapidly progressive AD (rpAD) (*n* = 15), VaD (*n* = 21), and cognitively unimpaired patients with vascular encephalopathy (VE) (*n* = 25) cases. In the study, cohort significant differences were detected in tau, p-tau, and Aβ1-42 (Aβ42) levels between AD and VaD patients, but not for the neuron-specific enolase (NSE), S100B protein, 14-3-3 and YKL-40. Differential tau, p-tau, and Aβ42 levels between AD and VaD were confirmed in the validation cohort, which additionally showed no differences between AD and rpAD, nor between VaD and VE. The evaluation of the biomarker performance in discrimination between AD and VaD patients revealed that the best diagnostic accuracy could be obtained when tau, p-tau, and Aβ42 were combined in form of Aβ42/p-tau (AUC 0.84–0.90, sensitivity 77–81%, specificity 80–93%) and (tau × p-tau)/Aβ42 ratio (AUC 0.83–0.87, sensitivity 73–81%, specificity 78–87%). Altogether, our studies provided neurodegenerative biomarker profiles in two cohorts of AD and VaD patients favoring the combination of CSF biomarker to differentiate between diseases.

## Introduction

Subcortical vascular encephalopathy (VE) is a major cause of vascular cognitive impairment leading to vascular dementia (VaD) (Moorhouse and Rockwood, 2008; Staekenborg et al., 2008). White matter lesions on brain imaging combined with clinical symptoms (including cognitive impairment, gait disturbance, and various focal neurological signs as well as incontinence) are characteristic (Baezner et al., 2003). Patients with VE are a heterogeneous entity (Roman et al., 1993), consisting of patients with and without dementia, partly with an abnormal Aβ-ratio, with and without inflammation.

In contrast, AD is neuropathologically characterized by the deposition of amyloid fibrils formed by amyloid beta (Aβ) as well as by neurofibrillary tangles which are composed of hyperphosphorylated tau protein (Serrano-Pozo et al., 2011). Heterogeneity regarding the clinical presentation and the disease course in AD is increasingly recognized. In the framework of our surveillance studies, we described a new subtype of AD, rapidly progressive AD (rpAD) (Schmidt et al., 2011, 2012). This subtype of AD can mimic the clinical course of Creutzfeldt-Jakob disease patients with a progressive cognitive decline (>6 mini-mental test points/year), short disease duration (<2 years, 6–8 month) as well as early focal neurological signs, such as occurrence of extrapyramidal symptoms and myoclonus. Patients with rpAD exhibit an age range between 60 and 70 years, whereas patients with classical AD are often older at disease onset (age range 70–80 years) and a disease duration of 7 years is acknowledged (Schmidt et al., 2012). A problem in the clinical setting arises because current diagnostic criteria (Roman et al., 1993; McKhann et al., 2011) often fail in discriminating AD and VaD leading to the diagnosis of mixed dementia. Even though both diseases exhibit the same vascular risk factors, such as atherosclerosis, diabetes mellitus, etc., AD and VaD/VE are different disease entities. Cases exhibiting a pathophysiologic overlap of AD and VaD are considered as mixed dementia (Jellinger and Attems, 2007). For a reliable early diagnosis and the differentiation of AD and VaD subtypes, biomarker diagnostics may be helpful to predict the disease course. At the moment, the subgroup definition to distinguish between AD and rpAD or between VE and VaD is based on clinical criteria and neuropsychological testing (Mini Mental State test), indicating a strong need for objective quantitative compared cerebrospinal fluid (CSF) biomarkers.

Until now some effort had already been undertaken to detect characteristic immune profiles or neurodegenerative marker proteins in CSF, serum or plasma from patients with AD or VaD (Andreasen et al., 1998; Lee et al., 2009; Paraskevas et al., 2009; Kaerst et al., 2013; Stoeck et al., 2014; Llorens et al., 2015; Schmitz et al., 2015). Even though a differential regulation of the immune system and classical AD biomarkers (tau, p-tau, Aβ42) could be observed in both diseases (Andreasen et al., 1998; Paraskevas et al., 2009; Schmitz et al., 2015), the reliability of accuracy of these makers discriminating AD from VaD was not comparable to other diagnostic test systems of dementia diseases, such as assays of 14-3-3, tau or misfolded protein aggregates (Cramm et al., 2015, 2016; Schmitz et al., 2016a,b). Therefore, although CSF tests may be useful in the differential diagnosis of AD from VaD, lower specificity of current biomarkers and disease heterogeneity impedes absolute discrimination in the differential diagnostic context (Andreasen et al., 1998; Nagga et al., 2002; Paraskevas et al., 2009).

Recent studies in our group on neurodegenerative marker profiles in AD and VaD were promising (Kaerst et al., 2013; Hermann et al., 2014) and lead us to the idea of the present study to proceed investigating the discrimination of VaD/VE from AD patients by the use of CSF biomarkers. Here, two independent patient cohorts were analyzed in parallel for the levels of classical AD CSF biomarkers total tau, p-tau, Aβ42. The neuronal damage markers 14-3-3 and neuron-specific enolase (NSE) and the inflammatory markers calcium binding protein B (S-100B) and YKL-40 were also quantified in the study cohort. Additionally, in our validation cohort we included a subgroup of AD called rapidly progressive AD (rpAD) and a group of patients with cerebral small vessel disease showing no relevant cognitive deficits called VE. The diagnostic accuracy was calculated for each marker separately as well as in combination.

## Materials and methods

### Samples

Cohort 1 consists of patients who underwent lumbar puncture for diagnostic purposes in the Clinical Dementia Center Göttingen and were included in this study after clinical and image-based diagnose of either AD [based on recent criteria ICD-10 definition for Alzheimer's disease (AD) F.00 G.30] or VaD (based on ICD 10 definition, F01 and NINDS-AIREN criteria (Roman et al., 1993). Patients with possible other neurodegenerative, neuroinflammatory and neoplastic diseases as well as patients with acute or subacute cerebral ischemia were excluded. The control group was composed of patients with either clinically or pathologically defined alternative diagnosis and included cases diagnosed with psychiatric disorders (psychosis, bipolar disorder, depression, and schizophrenia), epilepsy, autoimmune diseases, meningitis, alcohol abuse disorder, headache, vertigo, pain syndromes and alternative neurologic conditions. Control cases did not present biomarker profiles indicating the presence of a neurodegenerative disease and the presence of neurodegeneration was excluded in the follow-up clinical diagnostic process.

In Cohort 2, we included cases from a separate study on rpAD and cases from a study on cerebral small vessel disease (groups VE and VaD). Exclusion criteria were the same as mentioned above.

VaD diagnosis was based on ICD 10 definition (F01) and NINDS-AIREN criteria (Roman et al., 1993). VE diagnosis was based on neuroimaging (presence of white matter lesions on MRI T2, Flair, or CCT) and exclusion of inflammatory CNS disease. Patients showed signs of cerebral small vessel disease on MRI or CCT and scores of >2 points on the ARWMC scale (Wahlund et al., 2001). They did not match ICD-10 criteria for dementia and showed MMSE scores >27 points. Inflammatory CNS disease had been excluded by CSF analysis. Only in this validation cohort, presence of an AD-like biomarker signature associated with amyloid pathology was excluded for the VaD group using a CSF Aβ42/40 ratio cut-off, which has been suggested for the discrimination of AD from other forms of dementia (Lewczuk et al., 2004). No other biomarkers are currently available for a more accurate discrimination.

Classical AD patients were diagnosed according to the Dubois criteria (Dubois et al., 2007). rpAD patients were selected according to clinical presentation as reported previously by the German CJD surveillance group (Schmidt et al., 2011, 2012). Rapid progression was defined by a velocity of cognitive decline >6 pts/year on the Mini Mental Status Examination scale (Schmidt et al., 2011): Velocity of decline was calculated using linear regression (least square method) in accordance with Villemagne et al. (2013).

Since there is a lack of autopsy acceptances, a neuropathological examination was not possible. Subgroups were separated after initial research project inclusion but before consideration for this study. All tests were performed in the Neurochemistry Laboratory at the Department of Neurology, University Medical School, Göttingen (Germany). CSF was obtained by lumbar puncture and processed immediately. CSF was examined for standard parameters, such as cell count, proteins, and immunoglobulins in order to exclude neuroinflammatory disease. No patient showed elevated cell count or intrathecal IgG synthesis.

### CSF tests

Routine CSF analysis did not reveal any abnormalities. CSF tau levels were measured using a commercially available enzyme-linked immunosorbent assay (ELISA) kit according to the manufacturer's instructions. Total tau was measured using INNOTEST™ hTAU Ag; (Fujirebio), tau phosphorylated at Thr181 (p-tau) was analyzed using an ELISA kit (INNOTEST™ PHOSPHO-TAU(181 P); Fujirebio). The LIAISON® NSE was used for NSE quantification. The LIAISON SANGTEC® 100 was used for S100B detection. Levels of Aβ42 were measured with an ELISA kit (INNOTEST™ AMYLOID (1-42); Fujirebio). YKL-40 was measured using the MicroVue YKL-40 EIA ELISA kit from Quidel following manufacturer's instructions. Protein 14-3-3 was tested by western blot as previously described (Zerr et al., 1998; Schmitz et al., 2014).

### Statistical analysis

For two group comparisons, the Mann–Whitney test was used. In multiple comparisons, the Kruskal–Wallis test was used. Dunn's multiple comparison was used for *post hoc* analysis. *P*-values lower than 0.05 were considered significant. Statistical analyses and calculations of Area Under the Curve (AUC) and 95% confidence intervals from Receiver Operating Characteristic (ROC) areas were carried out using GraphPad-Prism 7 software. The best cut-off for value of a single biomarker or combination of biomarkers value was estimated based on the Youden index (Youden, 1950). For each biomarker or combination of biomarkers sensitivity and specificity values were calculated.

### Ethics

The present study was conducted according to the revised Declaration of Helsinki and Good Clinical Practice guidelines and has been approved by the local ethics committee in the University Medical Center, Göttingen (No. 9/6/08, 19/11/09, and 18/8/15). Written informed consent was given by all study participants or their legal next of kin.

## Results

### Determination of CSF biomarker profiles in patients with Alzheimer's disease and vascular dementia

**Figure 1:**
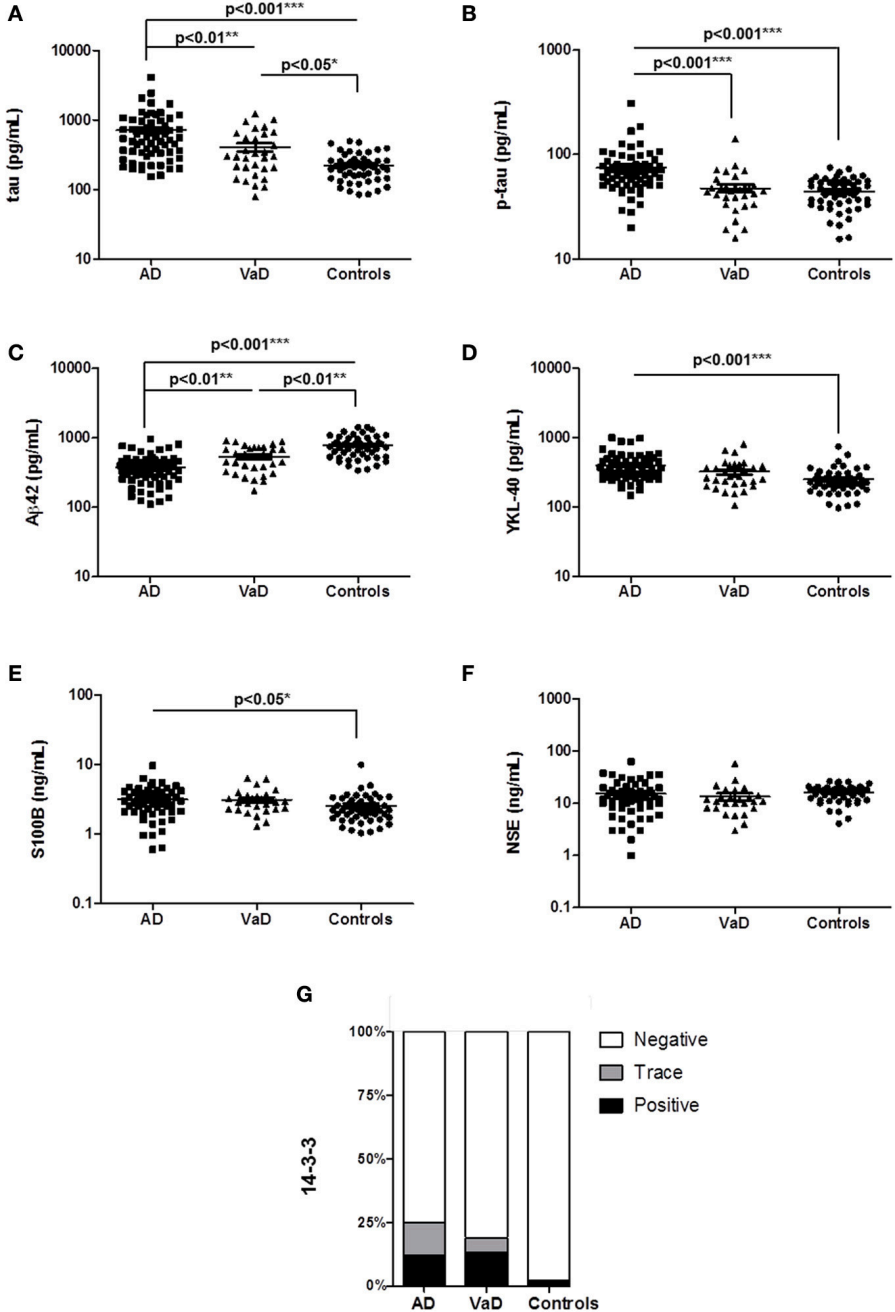
Determination of AD, inflammatory and neurodegenerative biomarker proteins in the CSF of AD, VaD, and control cases. **(A–F)** CSF levels of neurodegenerative biomarkers, total tau, *p*-tau, Aβ42, YKL-40, S100B, and NSE were measured by ELISA. **(G)** Semi-quantitative analysis of 14-3-3 levels. Patients analyzed by western blot were divided into three groups according to the 14-3-3 levels, which can be negative, trace (inconclusive) and positive. A *p* < 0.001 was considered as extremely significant (^***^), < 0.01 as very significant (^**^), < 0.05 as significant (^*^), and ≥ 0.05 as not significant.

In a study cohort of 50 controls, 65 AD, and 31 VaD accurately classified patients (without mixed pathology), we measured the CSF levels of total tau, p-tau, Aβ42, YKL-40, S100B, NSE, and 14-3-3 (Table 1A). Compared to controls, AD cases presented increased tau (*p* < 0.01), p-tau (*p* < 0.001), YKL-40 (*p* < 0.001), S100B (*p* < 0.05), and decreased Aβ42 (*p* < 0.001) levels (Figures 1A–E). In contrast, VaD only presented alterations on tau (*p* < 0.05) and Aβ42 levels (*p* < 0.01) (Figures 1A,C). Significant differences of total tau (*p* < 0.01), p-tau (*p* < 0.001) Aβ42 (*p* < 0.01) were detected between AD and VaD cases (Figures 1A–C).

Table 1Demographic and CSF biomarker data from study and validation cohorts.

**Table:** 

	**Control**	**AD**	**VaD**
**(A) STUDY COHORT-COHORT 1**			
**Demographics**			
*n*	50	65	31
Age (years)	70 ± 6	67 ± 11	70 ± 10
Gender (f/m)	27/23	43/22	27/6
**Biomarkers**			
tau (pg/mL)^*^	225 ± 103	718 ± 639	410 ± 300
p-tau (pg/mL)^*^	44 ± 14	77 ± 40	48 ± 23
Aβ42 (pg/mL)^*^	777 ± 276	378 ± 178	535 ± 219
YKL-40 (pg/mL)^*^	254 ± 117	400 ± 181	324 ± 156
NSE (ng/mL)^*^	16 ± 5	15 ± 11^§^	13 ± 11^§§^
S100B (ng/mL)^*^	2.5 ± 1.4	3.2 ± 1.9^§^	3.1 ± 1.2^§§^
14-3-3 (p–t–n) (%*p*)	1–0–49 (2%)	8–9–48 (12%)	4–2–25 (13%)

**Table:** 

	**AD**	**rpAD**	**VaD**	**VE**
**(B) VALIDATION COHORT-COHORT 2**				
**Demographics**				
*n*	26	15	21	25
Age (years)^*^	71 ± 11	68 ± 10	74 ± 7	74 ± 6
Gender (f/m)	16/10	11/4	13/8	16/9
**Biomarkers**				
tau (pg/mL)^*^	628 ± 456	700 ± 447	300 ± 176	301 ± 249
p-tau (pg/mL)^*^	100 ± 46	104 ± 71	59 ± 35	47 ± 23
Aβ42 (pg/mL)^*^	381 ± 99	401 ± 133	674 ± 292	875 ± 206

* *Mean values ± SD*,

§ 57 and

§§ *26 cases analyzed, p-t-n: positive, trace, negative, % p: % of positive cases*.

*Number of cases, age, and gender distribution as well CSF biomarker profile is reported*.

NSE levels were not different regulated between groups (Figure 1F). The percentage of 14-3-3 positive cases was higher in AD (12%) and VaD (13%) than in controls (2%) but not significantly different between AD and VaD patients (Figure 1G).

**Figure 2:**
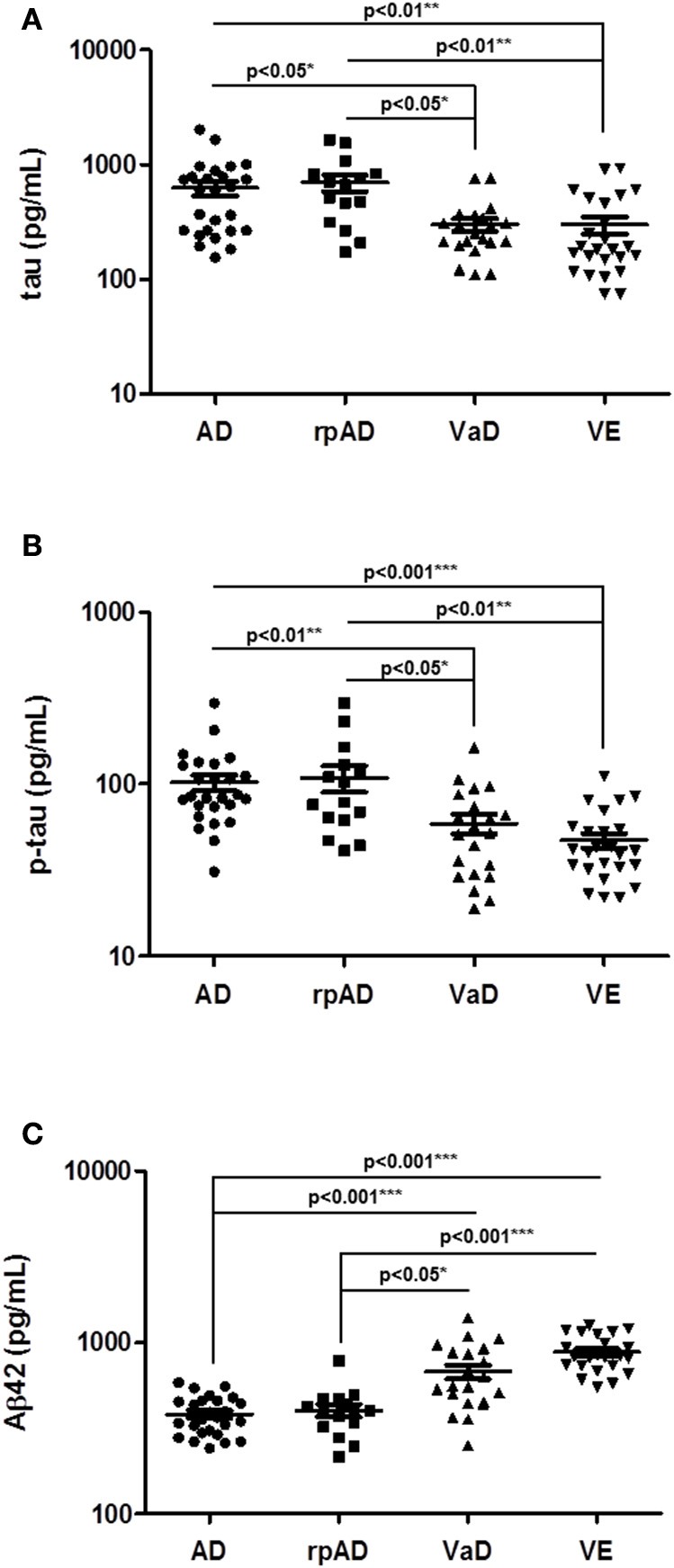
Determination of selected biomarker proteins in the CSF of a validation cohort of AD, rpAD, VaD, and VE cases. **(A–C)** CSF levels of total tau, p-tau, and Aβ42 were validated in a second cohort, consisting of AD, rpAD, VaD and VE patients. A *p* < 0.001 was considered as extremely significant (^***^), <0.01 as very significant (^**^), <0.05 as significant (^*^), and ≥0.05 as not significant (ns).

Subsequently, we validated our findings for total tau, p-tau, and Aβ42 in an independent second cohort of patients (Table 1B). The validation cohort included the rpAD subgroup and VE cases. A comparative analysis of four different patient groups (AD, rpAD, VaD, and VE) confirmed the main observations from the study cohort, since tau (*p* < 0.05), p-tau (*p* < 0.01), and Aβ42 (*p* < 0.001) were differentially regulated between AD and VaD samples (Figures 2A–C). Additionally, it could be shown that the subgroups rpAD and VE did not show any distinctive features when compared to AD or VaD (Figures 2A–C).

### Analysis of diagnostic accuracy of total tau, p-tau, and Aβ42 separately and in combination

At first, we calculated the diagnostic values of the biomarker proteins (CSF total tau, p-tau, and Aβ42), which were detected to be differently regulated between AD and VaD patients. Since no differences were detected in the biomarker profile between AD and rpAD subgroups, all AD cases were used for further analysis (*n* = 41). AUC, 95% confidence interval (CI) as well as cut-off and associated sensitivity and specificity values were calculated (Table 2). Sensitivity/specificity values showed relative poor accuracy for single biomarker measurements, tau, p-tau, and Aβ42 in the discrimination of both diseases, with AUC values ranging from 0.70 to 0.82 (Table 2). As reported before, a combination of different biomarker proteins may improve the diagnostic value and reliability of biomarker detection (Blennow et al., 2010; Llorens et al., 2016).

**Table 2:** CSF biomarker accuracy in the discrimination of AD from VaD cases in study and validation cohorts.

**Biomarker outcome**	**AUC**	**95% CI**	**Cut-off**	**Sensitivity (%)**	**Specificity (%)**
**STUDY COHORT-COHORT 1**					
AD: *n* = 65, VaD: *n* = 31				
p-tau	0.80	0.70–0.90	<51	77	81
Aβ42	0.70	0.59–0.81	>412	65	66
Aβ42/tau	0.79	0.69–0.88	>1	74	72
Aβ42/p-tau	0.84	0.75–0.93	>8.2	81	80
tau × p-tau/Aβ42	0.83	0.74–0.91	<48	73	78
**VALIDATION COHORT-COHORT 2**					
AD: *n* = 41, VaD: *n* = 21				
tau	0.74	0.63–0.87	<420	89	60
p-tau	0.78	0.65–0.90	<65	72	73
Aβ42	0.82	0.72–0.93	>490	72	88
Aβ42/tau	0.83	0.72–0.95	>1.2	81	73
Aβ42/p-tau	0.90	0.80–0.98	>8.4	77	93
tau × p-tau/Aβ42	0.87	0.77–0.97	<44	81	87

*AUC, 95% CI, cut-off value as well as sensitivity and specificity values are indicated for single biomarkers and combination of biomarkers*.

Therefore, we explored if the diagnostic accuracy may be improved by combining different biomarkers. Indeed, the combination of all three biomarkers in form of Aβ42/tau, Aβ42/p-tau, and tau × p-tau/Aβ42 revealed higher AUC values than single biomarker measurements. AUC values ranged from 0.79 to 0.90 (Table 2). Highest diagnostic accuracy in both cohorts was detected for Aβ42/p-tau ratio: AUC: 0.84, 95% CI: 0.75–0.93, sensitivity of 81% and a specificity of 80% in cohort 1 and AUC: 0.90, 95% CI: 0.80–0.98, sensitivity of 77% and a specificity of 93% in the validation cohort (Table 2, Supplementary Figure 1). Interestingly, cut-off values for combined biomarkers were similar between both cohorts (Table 2). For those biomarker ratios showing higher clinical accuracy we used cut-offs determined in the study cohort to test the diagnostic performance on the validation cohort. For Aβ42/p-tau, using an optimal cut off of 8.2 determined from study cohort, a sensitivity of 72% and a specificity of 95% was detected in the validation cohort. For tau × p-tau/Aβ42, using an optimal cut-off of 48 determined from study cohort, a sensitivity of 81% and a specificity of 85% was detected in validation cohort. This data indicates that parameters detected from the intra-cohort validation analysis were in range than those detected from the two cohorts individually (Table 2).

## Discussion

In patients with late-onset AD, approximately 24% of cases show a relevant vascular pathology in autopsy (Jellinger and Attems, 2007). Since vascular factors, such as diabetes or arteriosclerosis, belong to the risk factors of AD and a cerebral vascular pathology may further contribute to neurodegeneration in AD, both pathologies can occur to the same time, considered as mixed dementia. In these patients, an accurate classification remains problematic (de la Torre, 2002; Roman and Royall, 2004). Therefore, we excluded VaD patients showing a pathological amyloid β ratio from analysis. This was done in order to define a group of pure VaD, assigned as validation cohort, with no or minimal interference from mixed cases.

Given the clinical overlap between AD and VaD when using established diagnostic criteria, it is highly relevant to identify either imaging or biochemical biomarkers. Indeed, symptoms and pathophysiology often overlap and patients may experience similar cognitive, functional and behavioral changes (Roman and Royall, 2004). Previous studies often focused on the differentiation between AD and controls without neurodegeneration, but only few reports addressed the more challenging discrimination between AD and VaD patients (Andreasen et al., 1998; Nagga et al., 2002; Paraskevas et al., 2009; Kaerst et al., 2014; Skillback et al., 2015), most of them focusing on the classical CSF AD biomarkers. Therefore, the aim of this study was to extent the profiling of neurodegenerative marker-proteins in the CSF of AD and VaD patients because medical care plan and pharmaceutical treatments might be different for these two disease populations. In addition to classical markers in AD, such as total tau, p-tau, and Aβ42, we focused on alternative markers for neuronal degeneration for the first time, such as 14-3-3 (Sanchez-Juan et al., 2006; Schmitz et al., 2016b) and NSE (Zerr et al., 1995), both related to Creutzfeldt-Jakob disease due to massive neuronal degeneration, as well as S100B and YKL-40, two well-known markers of astrocytosis in neurodegenerative pathologies (Rothermundt et al., 2003; Bonneh-Barkay et al., 2010; Craig-Schapiro et al., 2010). In the validation cohort we also included the subgroups rpAD and VE with the aim to extend our analysis to different clinically relevant subgroups related to Alzheimer's and vascular pathology.

### Cerebrospinal fluid biomarkers in the discrimination of vascular dementia from Alzheimer's disease

The biomarker detection in CSF is a useful tool to measure any signs of neurodegeneration which are relevant in several dementia diseases. Our data indicated different CSF levels of total tau, p-tau (both elevated), and Aβ42 (decreased) in AD patients when compared to VaD cases and controls. Additionally, we found in VaD patients that CSF levels of p-tau were not significantly different from controls. These findings are in agreement with other reports (Andreasen et al., 1999, 2001; Nagga et al., 2002; Jia et al., 2005; Stefani et al., 2005; Paraskevas et al., 2009; Kaerst et al., 2013). The presence of elevated levels of the astrocytic marker YKL-40 in AD, but not in VaD, is also in line with previous reports (Janelidze et al., 2016). Interestingly S100B, another astrocytic marker of neuroinflammation (Donato et al., 2009), was elevated in AD cases compared to controls, which coincides with other studies (Peskind et al., 2001). The same could be observed in other neurological diseases associated to neuroinflammatory profiles (Michetti et al., 1979; Berger et al., 2002; Rejdak et al., 2008; Sussmuth et al., 2010). Altogether our data indicate that the astrocytic response and neuroinflammatory signature reported in AD pathology can be detected in the CSF of AD patients by quantification of astrocytic markers. This suggests that YKL-40 and S100B quantification could have an application in the evaluation of a potential therapeutic intervention in AD. The comparison of AD and VaD patients revealed no significant differences in YKL-40 and S100B nor in 14-3-3 and NSE levels.

Additionally, despite the increase in 14-3-3 positive cases in both diseases (compared to controls), 14-3-3 quantification does not have a clinical use in the diagnosis of AD and VaD due to its low sensitivity, which we confirmed from previous report (Blennow et al., 2010). Contrary to the previously reported increased NSE levels in AD and VaD cases (Blennow et al., 1994), we found similar NSE levels in controls, AD and VaD cases. Some explanations for these differences would be different types of controls used in both studies. Indeed, Blennow et al. used healthy individuals, while our control cohort is composed of cases with neurological alterations, which are more prone to present some kind of neuronal damage than healthy controls (Blennow et al., 1994).

This indicates that neuronal/axonal degeneration is not an appropriate pathologic hallmark for the discrimination of both diseases in CSF, in agreement with the partial overlap on tau levels, another reported marker of axonal damage, between AD and VaD. Besides the poor diagnostic accuracy of tau in the discrimination of AD from VaD, the observation of higher tau levels, but not of 14-3-3 and NSE in AD compared to VaD would suggest that, a specific AD mechanism, rather than axonal damage, contributes to elevated CSF tau in AD.

The validation of total tau, p-tau, and Aβ42 in a validation cohort of patients confirmed our observations and additionally revealed that AD biomarkers were not adequate to discriminate between AD and rpAD nor between VaD and VE. A lack of differences between both AD forms suggest the absence of differential pathology at the time of diagnosis because CSF tau, p-tau, and Aβ42 reflect pathophysiological processes of the brain correlating with amyloid plaques and neurofibrillary tangles (Tapiola et al., 1997, 2009). Additionally, our data indicate that tau, p-tau, and Aβ42 are not valuable markers predicting cognitive decline in clinically diagnosed AD patients.

The calculation of the diagnostic accuracy revealed sensitivity and specificity values varying between 62–89 and 60–88%, respectively for single biomarkers. These data are in agreement with Stefani et al., who suggested that Aβ42 alone showed a good discrimination between AD and VaD. The authors obtained sensitivity and specificity values of 77 and 80% (Stefani et al., 2005). In this regard, although study cohort could be considered more representative of the overall patient population (patients who underwent lumbar puncture for diagnostic purposes in our Clinical Dementia Center) than validation cohort (cases recruited for independent studies when AD or VaD diagnosis was suspected), overall diagnostic parameters were similar in both cohorts.

Before this study, there were only a few studies using the combination of different neurodegenerative markers to distinguish between AD and VaD (Andreasen et al., 1998; Paraskevas et al., 2009). Our results are in line with two other studies which suggested either the ratio of Aβ42/p-tau CSF levels or the combination of total tau, p-tau, and Aβ42 for a differentiation between AD and VaD patients. Both studies achieved discrimination with a sensitivity and specificity of 85% (Paraskevas et al., 2009). However, our results indicate that neither measurement of CSF tau, p-tau, or Aβ42 alone, nor the combination of these biomarkers can discriminate entirely between AD and VaD cases.

## Conclusions

In conclusion, our present study suggests that the combination of AD biomarkers especially Aβ42/p-tau and tau × p-tau/Aβ42 may be helpful for the discrimination of AD from VaD. Additional markers, such as YKL40, S100B, NSE, and 14-3-3 failed in distinguishing between diseases. For a further improvement of diagnostic accuracy, the identification of additional CSF biomarkers is required.

## Author contributions

FL, MS, and IZ conceived the study. FL, MS, and PL performed biomarker's measurements. FL and MS analyzed data. TK, CS, AF, and PH characterized patients and/or contributed samples/reagents. FL and MS drafted the manuscript. All authors interpreted the data, revised the manuscript for important intellectual content and read, and approved the final manuscript version.

### Conflict of interest statement

The authors declare that the research was conducted in the absence of any commercial or financial relationships that could be construed as a potential conflict of interest.

## Abbreviations

AD: Alzheimer's disease
Aβ42: amyloid beta 1–42 peptide
CSF: cerebrospinal fluid
ELISA: enzyme-linked immunosorbent assay
p-tau: phosphorylated tau
VE: vascular encephalopathy
VaD: vascular dementia.

## References

[B1] Andreasen N. Hesse C. Davidsson P. Minthon L. Wallin A. Winblad B.. (1999). Cerebrospinal fluid beta-amyloid(1-42) in Alzheimer disease: differences between early- and late-onset Alzheimer disease and stability during the course of disease. Arch. Neurol. 56, 673–680. 10.1001/archneur.56.6.673 10369305

[B2] Andreasen N. Minthon L. Davidsson P. Vanmechelen E. Vanderstichele H. Winblad B.. (2001). Evaluation of CSF-tau and CSF-Abeta42 as diagnostic markers for Alzheimer disease in clinical practice. Arch. Neurol. 58, 373–379. 10.1001/archneur.58.3.373 11255440

[B3] Andreasen N. Vanmechelen E. Van de Voorde A. Davidsson P. Hesse C. Tarvonen S.. (1998). Cerebrospinal fluid tau protein as a biochemical marker for Alzheimer's disease: a community based follow up study. J. Neurol. Neurosurg. Psychiatr. 64, 298–305. 10.1136/jnnp.64.3.298 9527138 PMC2170016

[B4] Baezner H. Daffertshofer M. Hennerici M. (2003). Subkortikale vaskuläre Enzephalopathie. Akt Neurol. 30, 266–280. 10.1055/s-2003-40905 14579623

[B5] Berger R. P. Pierce M. C. Wisniewski S. R. Adelson P. D. Clark R. S. Ruppel R. A.. (2002). Neuron-specific enolase and S100B in cerebrospinal fluid after severe traumatic brain injury in infants and children. Pediatrics 109:E31. 10.1542/peds.109.2.e31 11826241

[B6] Blennow K. Hampel H. Weiner M. Zetterberg H. (2010). Cerebrospinal fluid and plasma biomarkers in Alzheimer disease. Nat. Rev. Neurol. 6, 131–144. 10.1038/nrneurol.2010.4 20157306

[B7] Blennow K. Wallin A. Ekman R. (1994). Neuron specific enolase in cerebrospinal fluid: a biochemical marker for neuronal degeneration in dementia disorders? J. Neural Transm. Park. Dis. Dement. Sect. 8, 183–191. 10.1007/BF02260939 7748462

[B8] Bonneh-Barkay D. Wang G. Starkey A. Hamilton R. L. Wiley C. A. (2010). *In vivo* CHI3L1 (YKL-40) expression in astrocytes in acute and chronic neurological diseases. J. Neuroinflamm. 7:34. 10.1186/1742-2094-7-34 20540736 PMC2892443

[B9] Craig-Schapiro R. Perrin R. J. Roe C. M. Xiong C. Carter D. Cairns N. J.. (2010). YKL-40: a novel prognostic fluid biomarker for preclinical Alzheimer's disease. Biol. Psychiatry 68, 903–912. 10.1016/j.biopsych.2010.08.025 21035623 PMC3011944

[B10] Cramm M. Schmitz M. Karch A. Mitrova E. Kuhn F. Schroeder B.. (2016). Stability and reproducibility underscore utility of RT-QuIC for diagnosis of Creutzfeldt-Jakob disease. Mol. Neurobiol. 53, 1896–1904. 10.1007/s12035-015-9133-2 25823511 PMC4789202

[B11] Cramm M. Schmitz M. Karch A. Zafar S. Varges D. Mitrova E.. (2015). Characteristic CSF prion seeding efficiency in humans with prion diseases. Mol. Neurobiol. 51, 396–405. 10.1007/s12035-014-8709-6 24809690 PMC4309904

[B12] de la Torre J. C. (2002). Alzheimer disease as a vascular disorder: nosological evidence. Stroke 33, 1152–1162. 10.1161/01.STR.0000014421.15948.67 11935076

[B13] Donato R. Sorci G. Riuzzi F. Arcuri C. Bianchi R. Brozzi F.. (2009). S100B's double life: intracellular regulator and extracellular signal. Biochim. Biophys. Acta 1793, 1008–1022. 10.1016/j.bbamcr.2008.11.009 19110011

[B14] Dubois B. Feldman H. H. Jacova C. Dekosky S. T. Barberger-Gateau P. Cummings J.. (2007). Research criteria for the diagnosis of Alzheimer's disease: revising the NINCDS-ADRDA criteria. Lancet Neurol. 6, 734–746. 10.1016/S1474-4422(07)70178-3 17616482

[B15] Hermann P. Romero C. Schmidt C. Reis C. Zerr I. (2014). CSF biomarkers and neuropsychological profiles in patients with cerebral small-vessel disease. PLoS ONE 9:e105000. 10.1371/journal.pone.0105000 25147945 PMC4141759

[B16] Janelidze S. Hertze J. Zetterberg H. Landqvist Waldo M. Santillo A. Blennow K.. (2016). Cerebrospinal fluid neurogranin and YKL-40 as biomarkers of Alzheimer's disease. Ann. Clin. Transl. Neurol. 3, 12–20. 10.1002/acn3.266 26783546 PMC4704480

[B17] Jellinger K. A. Attems J. (2007). Neuropathological evaluation of mixed dementia. J. Neurol. Sci. 257, 80–87. 10.1016/j.jns.2007.01.045 17324442

[B18] Jia J. P. Meng R. Sun Y. X. Sun W. J. Ji X. M. Jia L. F. (2005). Cerebrospinal fluid tau, Abeta1-42 and inflammatory cytokines in patients with Alzheimer's disease and vascular dementia. Neurosci. Lett. 383, 12–16. 10.1016/j.neulet.2005.03.051 15936505

[B19] Kaerst L. Kuhlmann A. Wedekind D. Stoeck K. Lange P. Zerr I. (2013). Cerebrospinal fluid biomarkers in Alzheimer's disease, vascular dementia and ischemic stroke patients: a critical analysis. J. Neurol. 260, 2722–2727. 10.1007/s00415-013-7047-3 23877436 PMC3825487

[B20] Kaerst L. Kuhlmann A. Wedekind D. Stoeck K. Lange P. Zerr I. (2014). Using cerebrospinal fluid marker profiles in clinical diagnosis of dementia with Lewy bodies, Parkinson's disease, and Alzheimer's disease. J. Alzheimers Dis. 38, 63–73. 10.3233/JAD-130995 23948928

[B21] Lee K. S. Chung J. H. Choi T. K. Suh S. Y. Oh B. H. Hong C. H. (2009). Peripheral cytokines and chemokines in Alzheimer's disease. Dement. Geriatr. Cogn. Disord. 28, 281–287. 10.1159/000245156 19828948

[B22] Lewczuk P. Esselmann H. Otto M. Maler J. M. Henkel A. W. Henkel M. K.. (2004). Neurochemical diagnosis of Alzheimer's dementia by CSF Abeta42, Abeta42/Abeta40 ratio and total tau. Neurobiol. Aging 25, 273–281. 10.1016/S0197-4580(03)00086-1 15123331

[B23] Llorens F. Kruse N. Schmitz M. Shafiq M. da Cunha J. E. Gotzman N.. (2015). Quantification of CSF biomarkers using an electrochemiluminescence-based detection system in the differential diagnosis of AD and sCJD. J. Neurol. 262, 2305–2311. 10.1007/s00415-015-7837-x 26162713

[B24] Llorens F. Schmitz M. Karch A. Cramm M. Lange P. Gherib K.. (2016). Comparative analysis of cerebrospinal fluid biomarkers in the differential diagnosis of neurodegenerative dementia. Alzheimers Dement. 12, 577–589. 10.1016/j.jalz.2015.10.009 26718584

[B25] McKhann G. M. Knopman D. S. Chertkow H. Hyman B. T. Jack C. R. Jr. Kawas C. H.. (2011). The diagnosis of dementia due to Alzheimer's disease: recommendations from the national institute on aging-Alzheimer's association workgroups on diagnostic guidelines for Alzheimer's disease. Alzheimers Dement. 7, 263–269. 10.1016/j.jalz.2011.03.005 21514250 PMC3312024

[B26] Michetti F. Massaro A. Murazio M. (1979). The nervous system-specific S-100 antigen in cerebrospinal fluid of multiple sclerosis patients. Neurosci. Lett. 11, 171–175. 10.1016/0304-3940(79)90122-8 460686

[B27] Moorhouse P. Rockwood K. (2008). Vascular cognitive impairment: current concepts and clinical developments. Lancet Neurol. 7, 246–255. 10.1016/S1474-4422(08)70040-1 18275926

[B28] Nagga K. Gottfries J. Blennow K. Marcusson J. (2002). Cerebrospinal fluid phospho-tau, total tau and beta-amyloid(1-42) in the differentiation between Alzheimer's disease and vascular dementia. Dement. Geriatr. Cogn. Disord. 14, 183–190. 10.1159/000066023 12411760

[B29] Paraskevas G. P. Kapaki E. Papageorgiou S. G. Kalfakis N. Andreadou E. Zalonis I.. (2009). CSF biomarker profile and diagnostic value in vascular dementia. Eur. J. Neurol. 16, 205–211. 10.1111/j.1468-1331.2008.02387.x 19146641

[B30] Peskind E. R. Griffin W. S. Akama K. T. Raskind M. A. Van Eldik L. J. (2001). Cerebrospinal fluid S100B is elevated in the earlier stages of Alzheimer's disease. Neurochem. Int. 39, 409–413. 10.1016/S0197-0186(01)00048-1 11578776

[B31] Rejdak K. Petzold A. Stelmasiak Z. Giovannoni G. (2008). Cerebrospinal fluid brain specific proteins in relation to nitric oxide metabolites during relapse of multiple sclerosis. Mult. Scler. 14, 59–66. 10.1177/1352458507082061 17893112

[B32] Roman G. C. Royall D. R. (2004). A diagnostic dilemma: is “Alzheimer's dementia” Alzheimer's disease, vascular dementia, or both? Lancet Neurol. 3:141. 10.1016/S1474-4422(04)00674-X 14980525

[B33] Roman G. C. Tatemichi T. K. Erkinjuntti T. Cummings J. L. Masdeu J. C. Garcia J. H.. (1993). Vascular dementia: diagnostic criteria for research studies. Report of the NINDS-AIREN international workshop. Neurology 43, 250–260. 10.1212/WNL.43.2.250 8094895

[B34] Rothermundt M. Peters M. Prehn J. H. Arolt V. (2003). S100B in brain damage and neurodegeneration. Microsc. Res. Tech. 60, 614–632. 10.1002/jemt.10303 12645009

[B35] Sanchez-Juan P. Green A. Ladogana A. Cuadrado-Corrales N. Saanchez-Valle R. Mitrovaa E.. (2006). CSF tests in the differential diagnosis of Creutzfeldt-Jakob disease. Neurology 67, 637–643. 10.1212/01.wnl.0000230159.67128.00 16924018

[B36] Schmidt C. Haik S. Satoh K. Rabano A. Martinez-Martin P. Roeber S.. (2012). Rapidly progressive Alzheimer's disease: a multicenter update. J. Alzheimers Dis. 30, 751–756. 10.3233/JAD-2012-120007 22460329

[B37] Schmidt C. Wolff M. Weitz M. Bartlau T. Korth C. Zerr I. (2011). Rapidly progressive Alzheimer disease. Arch. Neurol. 68, 1124–1130. 10.1001/archneurol.2011.189 21911694

[B38] Schmitz M. Cramm M. Llorens F. Muller-Cramm D. Collins S. Atarashi R.. (2016a). The real-time quaking-induced conversion assay for detection of human prion disease and study of other protein misfolding diseases. Nat. Protoc. 11, 2233–2242. 10.1038/nprot.2016.120 27735933

[B39] Schmitz M. Ebert E. Stoeck K. Karch A. Collins S. Calero M.. (2016b). Validation of 14-3-3 protein as a marker in sporadic Creutzfeldt-Jakob disease diagnostic. Mol. Neurobiol. 53, 2189–2199. 10.1007/s12035-015-9167-5 25947081

[B40] Schmitz M. Hermann P. Oikonomou P. Stoeck K. Ebert E. Poliakova T.. (2015). Cytokine profiles and the role of cellular prion protein in patients with vascular dementia and vascular encephalopathy. Neurobiol. Aging 36, 2597–2606. 10.1016/j.neurobiolaging.2015.05.013 26170132

[B41] Schmitz M. Lullmann K. Zafar S. Ebert E. Wohlhage M. Oikonomou P. (2014). Association of prion protein genotype and scrapie prion protein type with cellular prion protein charge isoform profiles in cerebrospinal fluid of humans with sporadic or familial prion diseases. Neurobiol. Aging 35, 1177–1188. 10.1016/j.neurobiolaging.2013.11.010 24360565

[B42] Serrano-Pozo A. Frosch M. P. Masliah E. Hyman B. T. (2011). Neuropathological alterations in Alzheimer disease. Cold Spring Harb. Perspect. Med. 1:a006189. 10.1101/cshperspect.a006189 22229116 PMC3234452

[B43] Skillback T. Farahmand B. Y. Rosen C. Mattsson N. Nagga K. Kilander L.. (2015). Cerebrospinal fluid tau and amyloid-beta1-42 in patients with dementia. Brain 138(Pt 9), 2716–2731. 10.1093/brain/awv181 26133663

[B44] Staekenborg S. S. van Straaten E. C. van der Flier W. M. Lane R. Barkhof F. Scheltens P. (2008). Small vessel versus large vessel vascular dementia: risk factors and MRI findings. J. Neurol. 255, 1644–1651; discussion 813-4. 10.1007/s00415-008-0944-1 18677637

[B45] Stefani A. Bernardini S. Panella M. Pierantozzi M. Nuccetelli M. Koch G.. (2005). AD with subcortical white matter lesions and vascular dementia: CSF markers for differential diagnosis. J. Neurol. Sci. 237, 83–88. 10.1016/j.jns.2005.05.016 15990115

[B46] Stoeck K. Schmitz M. Ebert E. Schmidt C. Zerr I. (2014). Immune responses in rapidly progressive dementia: a comparative study of neuroinflammatory markers in Creutzfeldt-Jakob disease, Alzheimer's disease and multiple sclerosis. J. Neuroinflammation. 11:170. 10.1186/s12974-014-0170-y 25315814 PMC4207356

[B47] Sussmuth S. D. Sperfeld A. D. Hinz A. Brettschneider J. Endruhn S. Ludolph A. C.. (2010). CSF glial markers correlate with survival in amyotrophic lateral sclerosis. Neurology 74, 982–987. 10.1212/WNL.0b013e3181d5dc3b 20308682

[B48] Tapiola T. Alafuzoff I. Herukka S. K. Parkkinen L. Hartikainen P. Soininen H.. (2009). Cerebrospinal fluid β-amyloid 42 and tau proteins as biomarkers of Alzheimer-type pathologic changes in the brain. Arch. Neurol. 66, 382–389. 10.1001/archneurol.2008.596 19273758

[B49] Tapiola T. Overmyer M. Lehtovirta M. Helisalmi S. Ramberg J. Alafuzoff I.. (1997). The level of cerebrospinal fluid tau correlates with neurofibrillary tangles in Alzheimer's disease. Neuroreport 8, 3961–3963. 10.1097/00001756-199712220-00022 9462474

[B50] Villemagne V. L. Burnham S. Bourgeat P. Brown B. Ellis K. A. Salvado O.. (2013). Amyloid beta deposition, neurodegeneration, and cognitive decline in sporadic Alzheimer's disease: a prospective cohort study. Lancet Neurol. 12, 357–367. 10.1016/S1474-4422(13)70044-9 23477989

[B51] Wahlund L. O. Barkhof F. Fazekas F. Bronge L. Augustin M. Sjogren M.. (2001). A new rating scale for age-related white matter changes applicable to MRI and CT. Stroke 32, 1318–1322. 10.1161/01.STR.32.6.1318 11387493

[B52] Youden W. J. (1950). Index for rating diagnostic tests. Cancer 3, 32–35. 10.1002/1097-0142(1950)3:1<32::AID-CNCR2820030106>3.0.CO;2-3 15405679

[B53] Zerr I. Bodemer M. Gefeller O. Otto M. Poser S. Wiltfang J.. (1998). Detection of 14-3-3 protein in the cerebrospinal fluid supports the diagnosis of Creutzfeldt-Jakob disease. Ann. Neurol. 43, 32–40. 10.1002/ana.410430109 9450766

[B54] Zerr I. Bodemer M. Racker S. Grosche S. Poser S. Kretzschmar H. A.. (1995). Cerebrospinal fluid concentration of neuron-specific enolase in diagnosis of Creutzfeldt-Jakob disease. Lancet 345, 1609–1610. 10.1016/S0140-6736(95)90118-3 7783539

