# Supplementary material for: Cerebrospinal Fluid Biomarkers of Alzheimer's Disease Show Different but Partially Overlapping Profile Compared to Vascular Dementia
Source: Front Aging Neurosci. 2017 Sep 12;9:289. doi: 10.3389/fnagi.2017.00289 (PMC5601075; doi:10.3389/fnagi.2017.00289)
Supplement: Supplementary file 1 [file DataSheet1.docx]

**Supplementary Figure 1.** Determination of AB42/tau ratio in AD and VaD cases from study and validation cohorts. A p-value: < 0.001 was considered as extremely significant (***)
